# Sexual Violence Against Men: A Retrospective Study on Victim Characteristics, Violence Severity, and Occurrence of Injuries Among Male Victims Attending a Sexual Assault Center Between 2015 and 2022 in Stockholm, Sweden

**DOI:** 10.1177/08862605251361127

**Published:** 2025-08-27

**Authors:** Frida M. Larsson, Anna Nielsen, Zangin Zeebari, Mariano Salazar, Anna-Mia Ekström, Anna Möller

**Affiliations:** 1 Department of Global Public Health, Karolinska Institute, Stockholm, Sweden; 2 Jönköping International Business School (JIBS), Jönköping University, Sweden; 3 Department of Clinical Science and Education, Södersjukhuset, Karolinska Institute, Stockholm, Sweden; 4 Department of Obstetrics and Gynecology, Södersjukhuset, Stockholm, Sweden

**Keywords:** rape, male victims, NorVold Abuse Questionnaire, severity of violence, and injury

## Abstract

Research on sexual violence often overlooks men, with limited studies focusing on male victims. A deeper understanding of this issue is essential for providing evidence-based healthcare and effective support for male victims. Therefore, this study aims to (a) describe the characteristics of sexual violence among male victims seeking emergency care at Sweden’s largest sexual assault center and (b) examine whether the occurrence of injuries and the severity of violence differ according to victim characteristics, assault characteristics, and the victim’s relationship to the assailant. This retrospective study analyzed 245 anonymized medical and forensic records of men who visited Stockholm’s sexual assault center, Sweden, from 2015 to 2022. The severity of violence was assessed with an adapted NorVold Abuse Questionnaire. Descriptive and inferential statistics were used to analyze the data. In our dataset, 92% of victims experienced severe acts of sexual violence, and 27% faced severe physical violence during the assault. Assaults by a group were associated with increased severity of physical violence in the adjusted model (adjusted odds ratio [AOR] = 3.9, 95% CI [1.2, 12.5]). Additionally, 65% of victims sustained extragenital injuries, which were linked to being assaulted by a known assailant (AOR = 5.8 [1.4, 24.9]), the victim being under the influence of substances during the assault (AOR = 2.5 [1.0, 6.4]), and exposure to moderate/severe physical violence (AOR = 6.6 [2.5, 17.1]). Regarding the victim’s mental health history, 24% reported having a neuropsychiatric diagnosis and 48% reported a psychiatric disorder. Additionally, 45% had a history of prior sexual assault. Our study suggests that the men who sought post-assault care frequently reported experiencing physical violence during the sexual assault, particularly in cases involving multiple assailants. The high prevalence of self-reported mental illness, neuropsychiatric diagnoses, and prior sexual assault among these individuals underscores the need for psychosocial support for this patient group.

## Introduction

Male victims of sexual violence are often overlooked, both in research and in clinical practice (Hequembourg et al., 2015; Peterson et al., 2011; Thomas & Kopel, 2023; Zilkens et al., 2018). However, like women, men can be exposed to unwanted sexual acts as a one-time event or part of systematic violence, which can lead to adverse psychological, physical, sexual, and social consequences. The literature even suggests that men experience greater health consequences compared to their female counterparts (Peterson et al., 2011).

Estimating the prevalence of male victimization is challenging due to men’s unwillingness to disclose their experiences and methodological and definitional differences across studies (Dworkin et al., 2021; Thomas & Kopel, 2023). Despite these barriers, national studies provide valuable insights into the prevalence of male victimization. For instance, in a population-based study from Sweden, 4.5% of men reported experiencing any sexual violence at some point in adulthood, and 6.5% of men had experienced more severe forms of sexual assault over their lifetime (Öberg et al., 2020). Another national survey showed that 1% of men had been subjected to forced intercourse through physical violence, and 9% had experienced other types of sexual assault, including unwanted kissing and touching (The Swedish Public Health Agency, 2019). Additionally, the Swedish National Council for Crime Prevention reported that in 2022, 1.2% of men aged 16 to 84 had been victims of a sexual offense, with the highest prevalence (4.4%) observed among young men aged 20 to 24 (Holst et al., 2023). In addition to young age, male sexual assault victimization is associated with being a sexual minority, having a neuropsychiatric diagnosis, using alcohol and substances, experiencing revictimization, and having adverse childhood experiences (Ghirardi et al., 2023; Ports et al., 2016; The Swedish Public Health Agency, 2019; Thomas & Kopel, 2023; Walker et al., 2019).

Furthermore, male victims are less likely to report sexual victimization to authorities, seek healthcare, and talk to relatives about their experiences. Feelings of shame and guilt, traditional masculinity norms, and rape myths that invalidate male experiences of sexual violence are described as barriers to disclosure (Monk-Turner & Light, 2010; Sorsoli et al., 2008; The Swedish Public Health Agency, 2019; Thomas & Kopel, 2023). Nevertheless, the aid, support, and resources for male victims are estimated to be over two decades behind those for female victims. Consequently, the understanding of male sexual victimization is significantly less comprehensive compared to the knowledge base on female victimization (Thomas & Kopel, 2023). Some international studies have contributed to the existing research on the characteristics of male victimization using data from sexual assault centers (Covers et al., 2022; Hiquet & Gromb-Monnoyeur, 2013; Kane et al., 2024; Larsen & Hilden, 2016; Zilkens et al., 2018), however, none from a Swedish context. An increased understanding of sexual violence against men, the assault characteristics, and the severity of the assaults is highly needed in order to gain a deeper understanding of this overlooked group and to inform and enable the provision of adequate and quality healthcare tailored for male victims. Therefore, this study aims to (a) describe the characteristics of sexual violence among male victims who have sought care at a sexual assault center in Stockholm, Sweden, and (b) examine whether the occurrence of injuries and the severity of violence differ according to victim’s characteristics, assault characteristics, and the relationship of the victim to the assailant. Our results will provide valuable insights into the patterns of violence and injury observed in male victims who seek healthcare following sexual violence.

## Methods

### Population and Study Setting

This retrospective study was conducted at the Emergency Clinic for Rape Victims at Södersjukhuset (South General Hospital) in Stockholm, Sweden. In the Stockholm Region, all healthcare for victims of sexual violence is centralized at this specialized clinic, which offers comprehensive care through medical assessments, forensic examinations, and support services. Post-assault care in Stockholm is organized so that if someone seeks help at any other clinic, such as a primary healthcare clinic, psychiatric clinic, or sexual health clinic, within 30 days of the assault, they are directed to the sexual assault center. Nevertheless, victims can choose to seek care at any regular emergency department in Stockholm after a sexual assault, though most ultimately visit the sexual assault center. The Emergency Clinic for Rape Victims serves residents of the Stockholm Region, visitors assaulted throughout Stockholm County, and individuals who are assaulted on the route to Stockholm. An interdisciplinary team, consisting of doctors, forensic nurses, and psychologists, is employed at the clinic. During the first 10 years (2005–2015), the clinic only attended to female victims, but since 2015, individuals of any gender can seek care at the clinic within 30 days of a sexual assault. Between January 1, 2015 and December 31, 2022, 6,437 victims sought emergency care after sexual violence at the clinic: 321 (5%) males and 6,116 (95%) females. During the emergency visit, all victims participated in a standardized interview conducted by either a forensic nurse or a doctor as part of the routine care. They were also offered a forensic examination conducted by the doctor, although not all consented to participate. The information from the interviews and the findings from the forensic examination were structurally written down in the patient’s medical record.

### Data Collection

In this study, medical and forensic records between 2015 and 2022 were selected based on the individual’s Social Security number, where a specific digit indicates their legal gender (male or female). This includes individuals with a transgender identity, identified through the free text sections in the medical records. Given the variation (transwomen and transmen) and incompleteness of data in the medical records for the transgender subgroup, these individuals are only included in the description of participant characteristics but excluded from the main data analysis.

The first author reviewed all anonymized patient records with a male-specific Social Security number and managed the variables, using the Electronic Data Capture tool REDCap hosted at Karolinska Institute (Harris et al., 2009, 2019). In instances of ambiguities during the data entry process, specific cases were discussed with the last author, a senior medical doctor at the clinic.

### Definitions

Background characteristics: gender (males only), age (continuous), relationship status (single/partner), living situation (alone/with parents/partner/other cohousing/homeless/other), and occupation (working/studying/unemployed/on sick leave/other). The option “other” for occupation included practical training, retirement, and daily activity for people with functional impairment. The variable “other” for the living situation was, for example, imprisonment, homes organized by the social services (such as protection shelters, homes for care or accommodation, and foster homes), and homes within The Swedish Act concerning Support and Service for Persons with Certain Functional Impairments.

Variables related to participants’ history of self-reported mental illness, functional impairment, substance abuse, and sexual trauma were assessed. Past or present psychiatric disorders (affective disorder, anxiety disorder, eating disorder, psychotic illness, posttraumatic stress disorder [PTSD], self-harm, suicidal thoughts or attempts, and others), neuropsychiatric diagnoses (Attention-Deficit/Hyperactivity Disorder (ADHD) and autism spectrum disorder), and functional impairments (mobility impairment, hearing and visual impairment, intellectual disability, and others) were coded as yes/no. Self-reported substance abuse of alcohol or illegal drugs (past/ongoing/no) and history of previous sexual violence (yes/no) were also assessed during the forensic interview.

The types of sexual assault were defined as vaginal, anal, or oral penetration with a penis, other body part, or object (both performing and receiving), genital touching, sexual touching of body parts (excluding genitals), and other forms of sexual violence. The variable “other” included acts such as verbal sexual harassment, oral contact with mouth and skin (excluding genitals), attempted rape, and sexual humiliation (e.g., filming/photographing the victim, being forced to look at pornography, being present during the sexual act, and being urinated at).

The relationship between victim and assailant was divided into two main groups: known assailants (including partner or ex-partner, close friend or acquaintance, new or superficial acquaintance, and family or relatives) and a stranger (no relationship between the victim and assailant). Additionally, the number of assailants (single/group), the ability to recall the assailant (yes/no), and the gender of the assailant (man/woman/other) were included.

The site of the sexual assault included the home environment, outdoor settings (both planned meetings and unexpected attacks), and other places (such as restaurants, bars, clubs, means of transportation, hotels, spas, massage salons, prisons/custody, and workplaces). The victim’s influence of substances during the event (yes/no), reporting the event to the police (yes/no), and time elapsed from the assault until attending the clinic (<72 hr/≥72 hr) were also investigated.

The physical violence performed during the assault was defined as holding, hitting, slapping, kicking, strangulation/suffocation attempt, pushing, use of or threat with a weapon, and other acts (such as biting, scratching, and hairpulling). Multiple violence was defined as the presence of two or more physical acts during the assault (yes/no).

The NorVold Abuse Questionnaire (NorAQ) was used to classify the severity of the sexual violence and physical violence used during the assault (I. M. K. Swahnberg & Wijma, 2003; K. Swahnberg, 2011). The NorAQ is a validated instrument tested in a Swedish context, that classifies violent acts into mild, moderate, and severe violence according to certain premises (I. M. K. Swahnberg & Wijma, 2003; K. Swahnberg, 2011). For this study, an adapted version of NorAQ was developed and used to assess the level of severity of the sexual assault and the physical violence performed during the assault (Supplemental Appendix 1: adapted classification of mild, moderate, and severe sexual and physical violence based on NorAQ). The main modification was that two or more mild violent acts during the assault were classified as moderate. Similarly, if the victim was exposed to several types of moderate violent acts, the violence was classified as severe.

The doctor assessed and documented the injuries in the patient’s records following the forensic examination. The definition of injuries included observation of bruises, redness, tears, or swelling on extragenital locations (head or neck, trunk, and extremities), anal locations (perineum, anus, and rectum), and genital locations (glans, preputium, frenulum, penis, and scrotum). Multiple injuries (yes/no) were classified as injuries on more than one body part.

### Sample Size and Statistical Analyses

**Figure 1.**
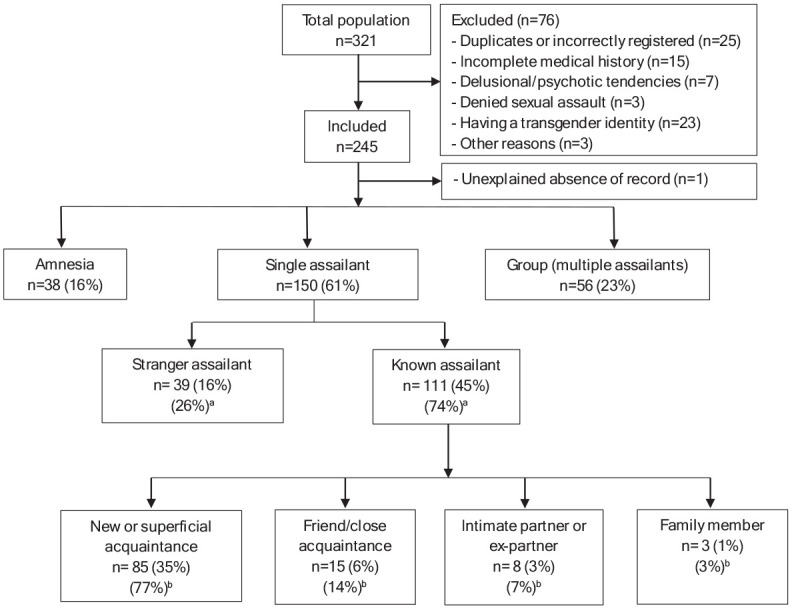
Flow diagram of the sampling and relationship between victim and assailant. *Note.* The percentage of victim’s relationship to the assailant is based on all included male victims. ^a^Percentage of single assailants. ^b^Percentage of known assailants.

A total number of 321 records of victims with a male Social Security number were reviewed. Individuals identified as transgender (*n* = 23) were excluded from the main analysis due to incomplete data (e.g., missing transgender people with a female Social Security number). Additional reasons for exclusion are provided in Figure 1.

This resulted in a final sample of 245 records, including 12 victims who visited the clinic several times (a total of 32 visits). Of these recurring victims, nine had visited the clinic twice. The results from the random effects model, which considers the dependence between repeated visits, were similar to the analysis when repeated visits were not considered; hence, all records (including the repeated visits) were included in the final analysis without random effects.

Pearson’s chi-squared test was employed to analyze categorical data, and an independent *t*-test was used for continuous data. Fisher’s exact test was applied if the data did not pass the assumptions for Pearson’s chi-squared test (i.e., if any expected cell frequency was less than 5).

To assess factors associated with our three main outcomes, the severity of physical violence, extra genital injuries, and anal injuries, binary logistic regression was used. Firstly, crude associations were studied, which examine the associations between a factor variable and an outcome variable without controlling for potential confounding factors. Secondly, a multivariable model was utilized to study the possible confounding effects of other variables in the model. The associations between the factor variable and the outcome variable were measured as odds ratio (OR) with 95% confidence intervals (CI). The results were considered statistically significant if the *p*-value was <.05.

Details on the following outcome variables were missing: severity of physical violence (26% missing), extragenital injuries (19% missing), and anal injuries (34% missing). Therefore, a multiple imputation analysis for sensitivity using the fully conditional specification method in SPSS was conducted. Since the results of the multiple imputation analysis were mostly consistent with the results of the complete case analysis, the latter was used in the final analysis (see Supplemental Appendices 2, 3, and 4). IBM SPSS Statistics version 29.0.1 was used to perform the analyses.

### Ethical Considerations

Ethical clearance was received in March 2022 from the Swedish Ethical Review Authority (ref. no: 2022-00170-01). To minimize the intrusion on the patient’s autonomy and privacy, all patient records were anonymized by the Department of Data Protection at Södersjukhuset before being received by the researchers.

## Results

### Victim Characteristics

The mean age of the male victims was 28.8 years (median 26 years, range 13–75), and 10.2% were minors (under 18 years of age). The majority of the victims presented to the clinic within 72 hrs of the sexual assault (69%). Everyone participated in the forensic interview; however, some did not consent to extragenital, genital, or anal examination (19%, 40%, and 34%, respectively). Additionally, 61% of the male victims in this study had reported the assault to the police. Regarding mental health history, almost half of the male victims reported having a psychiatric disorder (48%), and about one-fourth had a self-reported neuropsychiatric diagnosis (24%). In addition, just under half of the victims had a history of previous sexual assault (45%). A psychiatric disorder was reported by 70% of individuals with a history of sexual violence, compared to 30% of those without prior exposure to sexual assault (*p* < .001). When comparing the background characteristics of male victims and victims with transgender identity, the results showed a significantly higher prevalence of neuropsychiatric diagnosis and previous sexual assault among transgender individuals. See Table 1 for more information about the participant’s background characteristics, mental health, and sexual trauma experiences.

**Table 1.** Background Characteristics of Male and Transgender Victims Seeking Care at a Sexual Assault Center in Stockholm, Sweden from 2015 to 2022.

Victim’s Background Characteristics, Mental Health, and Trauma Experiences	Male Victims,* *n* = 245 (91%)	Transgender Victims,** *n* = 23 (9%)	Male vs. Transpersons, *p* > .05
Age (mean)	28.8 (±10.6)	28.8 (±11.6)	.981^a^
Relationship status			
Partner	56/226 (25%)	3/20 (15%)	.421^b^
Single	170/226 (75%)	17/20 (85%)	
Occupation			
Working/studying	151/223 (68%)	15/23 (65%)	.808^a^
Unemployed/sick leave/other	72/223 (32%)	8/23 (35%)	
Living situation			
Alone	85/236 (36%)	8/23 (35%)	.919^b^
Cohousing***	106/236 (45%)	10/23 (43%)	
Homeless/other	45/236 (19%)	5/23 (22%)	
Self-reported neuropsychiatric diagnosis			
Yes	56/230 (24%)	11/22 (50%)	.009^a^
No	174/230 (76%)	11/22 (50%)	
Self-reported psychiatric disorder			
Yes	107/223 (48%)	15/22 (68%)	.071^a^
No	116/223 (52%)	7/22 (32%)	
Functional impairment			
Yes	15/240 (6%)	3/23 (13%)	.200^b^
No	225/240 (94%)	20/23 (87%)	
Substance abuse (previous and present)			
Yes	36/228 (16%)	3/22 (14%)	1.00^b^
No	192/228 (84%)	19/22 (86%)	
History of sexual assault			
Yes	100/220 (45%)	18/23 (78%)	.003^a^
No	120/220 (55%)	5/23 (22%)	

a Pearson χ^2^-test for categorical data (column percentage), and an independent *t*-test for continuous data.

b Fisherman’s exact test.

* Male victims, include men (born male and identified as men).

**Transgender victims, include participants with transgender identity.

*** Including partner, parents, or another person.

### Relationship Between the Victim and Assailant

The majority of the victims described assaults committed by a single assailant (61%), of which, 74% of the assailants were known to the victim and 26% were strangers. Furthermore, 23% of the victims were assaulted by a group of assailants, and 16% experienced amnesia during the sexual assault and could not recall the assailant’s identity (Figure 1). Moreover, 88% (181/206) of the assailants were men, 10% (20/206) were women, and 2% (5/206) were categorized as “other” (men and women (group) *n* = 4 and transperson *n* = 1).

### Sexual Assault Characteristics

**Figure 2.**
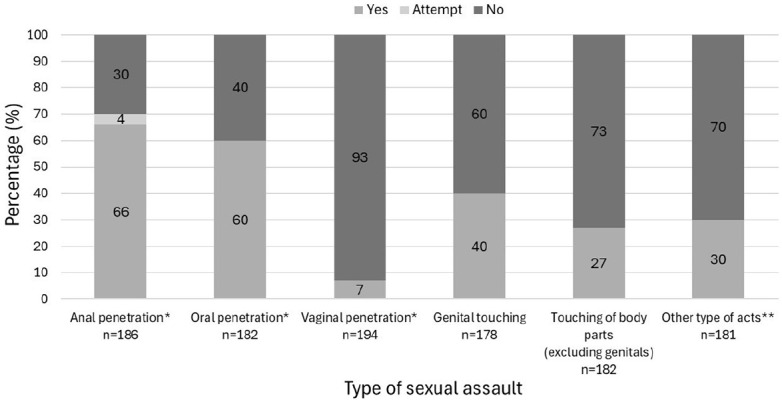
Type of sexual assaults reported among male victims seeking care at a sexual assault center in Stockholm, Sweden, between 2015 and 2022. *Note.* Unrecalled acts were excluded from the analysis due to memory-related missingness. *Was penetrated against their will and forced to penetrate another individual. **Other types of acts include: filming or photographing the victim, forced to look at pornography, verbal sexual harassment, ejaculation toward the victim’s face, spitting in the victim’s mouth, urination or defecation on the victim, use of sex toys, licking or oral contact with the victim’s skin, mouth or anus, the victim was present during a sexual act, being sexually looked at, and genital rubbing.

Anal penetration was the most common type of sexual assault (66%), of which almost all had received anal penetration by the assailant (98%). A few of the victims also experienced attempted anal penetration (4%), meaning penetration without successful completion by the assailant. Oral penetration was also commonly reported by the victims (60%), where 74% of the victims were nonconsensually penetrated orally, and 41% were forced to penetrate the assailant orally (some were forced to both give and receive oral penetration). Additionally, 7% were forced to penetrate a female assailant vaginally. Genital touching, touching of body parts (excluding genitals), and other types of acts were experienced by 40%, 27%, and 30%, respectively (see Figure 2 for more information). The majority of the sexual acts performed were classified as severe (92%), followed by moderate (6%), and mild (2%).

Some of the victims demonstrated acts of self-defense, either verbally (21%), physically (9%), or by a combination of both (30%). The assault took either place in a home environment (54%, *n* = 121/223), outdoors (16%, *n* = 35/223), or in another place (club/bar, taxi/car, cruise/ferry/boat, hotel room, spa/massage salon, prison, or workplace) (30%, *n* = 67/223). Half of the victims were under the influence of alcohol during the time of the sexual assault (50%, *n* = 117/233), and 10% (23/234) were under the influence of drugs. However, the quantity of substances was not considered. Additionally, 12% (27/234) believed they had been drugged in relation to the sexual assault.

Assaults committed by a single assailant were more likely to involve genital touching (46% [59/128] vs. 27% [13/49], *p* = .018) and occur in a home environment (60% [88/147] vs. 36% [20/55], *p* < .001) compared to those committed by a group of assailants. The results also showed a higher prevalence of victims being under the influence of substances (alcohol, drugs, suspected drugging) during assaults committed by a stranger compared to those committed by a known assailant (72% [26/36] vs. 50% [54/108], *p* = .02). No significant difference was found between substance use during the assault and the number of assailants. Additionally, the victims in group assaults showed a trend toward being of a higher age compared to assaults perpetrated by a single assailant (31.7 years vs. 28.5 years), although not significantly, *p* = .086. Otherwise, no differences were found when comparing assailant characteristics with the type of sexual assault, the severity level of the sexual violence, reporting to the police, or time lapse between assault and examination.

### Physical Violence During the Sexual Assault

Over half of the participants had been subjected to some form of physical violence during the sexual assault (71%). Holding (64%) was the most prevalent type of physical act, followed by hitting (26%), strangulation/suffocation attempts (16%), and “other” forms of physical violence such as biting, scratching, and hairpulling (15%). See Table 2 for more information. Additionally, 10% (19/184) of the victims reported threats and/or use of knives or guns during the assault (not included in the table). Moreover, 27% of the physical violence was classified as severe, 17% as moderate, and 27% as mild. Additionally, there was no physical violence involved in 29% of the assaults.

**Table 2.** Differences in Physical Violence During the Assault Based on the Relationship to the Assailant Among Male Victims Seeking Care at a Sexual Assault Center in Stockholm, Sweden, from 2015 to 2022.

	Total Exposure to Physical Violence	Number of Assailants			Relationship Between Victim and Assailant (Single Assaults)		
	Total (*n* = 245), *n* (%)	Single (*n* = 150), *n* (%)	Group (*n* = 56), *n* (%)	*p* < .05	Known (*n* = 111), *n* (%)	Stranger (*n* = 39), *n* (%)	*p* < .05
Type of physical violence							
Any physical violence	129/182 (71)	82/130 (63)	47/51 (92)	<.001^a^	63/98 (64)	19/32 (59)	.617^a^
Holding	115/181 (64)	74/130 (57)	41/50 (82)	.002^a^	56/98 (57)	18/32 (56)	.929^a^
Hitting	47/181 (26)	24/130 (18)	23/50 (46)	<.001^a^	21/98 (21)	3/32 (9)	.127^a^
Slapping	16/47 (34)	—	—	—	—	—	—
Fists	10/47 (21)	—	—	—	—	—	—
Object	4/47 (9)	—	—	—	—	—	—
Unspecified	17/47 (36)	—	—	—	—	—	—
Kicking	14/179 (8)	5/129 (4)	9/49 (18)	.003^b^	3/97 (3)	2/32 (6)	.597^b^
Strangulation/suffocation attempt	28/178 (16)	19/129 (15)	9/48 (19)	.515^a^	17/97 (18)	2/32 (6)	.155^b^
Pushing	25/182 (14)	14/130 (11)	11/51 (22)	.058^a^	11/98 (11)	3/32 (9)	1.0^b^
Other physical acts	27/180 (15)	17/130 (13)	10/49 (20)	.222^a^	13/98 (13)	4/32 (13)	1.0^b^
Multiple physical acts	74/181 (41)	44/130 (34)	30/50 (60)	.001^a^	35/98 (36)	9/32 (28)	.431^a^
Severity of physical violence							
None/mild	101/181 (56)	82/130 (63)	18/50 (36)	.001^a^	61/98 (62)	21/32 (66)	.731^a^
Moderate/severe	80/181 (44)	48/130 (37)	32/50 (64)		37/98 (38)	11/32 (34)	

*Note.* Unrecalled acts were excluded from the analysis due to memory-related missingness.

a Pearson χ^2^-test for categorical data (column percentage).

b Fisherman’s exact test.

The prevalence of any physical violence was significantly higher in assaults perpetrated by a group compared to assaults committed by a single assailant (92% vs. 63%, *p* < .001). The victims in group assaults were also significantly more often exposed to moderate/severe physical violence compared to assault with a single perpetrator (64% vs. 37%, *p* = .001). No significant differences were found regarding the prevalence of physical violence between assaults perpetrated by known and stranger assailants (Table 2).

In the crude regression analysis, factors associated with increased odds of moderate/severe physical violence during the sexual assault were victims being of older age (≥30 years of age) (OR = 2.3, 95% CI [1.0, 5.3]) and being assaulted by a group of assailants (OR = 3.4 [1.3, 8.6]). After adjusting for other variables in the multivariable model, the only remaining significant factor associated with an increased severity level of physical violence was being assaulted by a group (adjusted OR = 3.9 [1.2, 12.5]).

### Injury Characteristics

Over half of the participants had extragenital injuries (65%) on the head/neck (35%), trunk (33%), and extremities (55%). Additionally, 3% had genital injuries and 36% had anal injuries. Multiple injuries (different locations) were present in 44% of the cases. The factors associated with extragenital injuries are presented in Table 3. The crude analysis resulted in a positive association between extragenital injuries and being assaulted by a group (OR = 3.0, 95% CI [1.1, 7.9]) and being an adult (OR = 2.4 [1.1, 5.4]); however, these associations did not remain significant in the multivariable analysis. After adjusting for all factors in the model, being assaulted by a known assailant, being under the influence of substances during the sexual assault, and being exposed to moderate/severe physical violence were related to the presence of extragenital injuries. Furthermore, no factors were significantly associated with anal injury.

**Table 3.** Factors Associated with Extragenital Injuries After Sexual Assault Among Male Victims Seeking Care at a Sexual Assault Center in Stockholm, Sweden, Between 2015 and 2022.

Variable	Level	*n*	%^a^	Crude Analysis, OR [95% CI]	Multivariable Analysis,^b^ AOR [95% CI]
Age	Adolescence (aged 13–19)	21	16	Reference	Reference
	Young adults (aged 20–29)	56	43	1.5 [0.7, 3.2]	1.0 [0.3, 3.2]
	Adults (aged 30+)	53	41	2.4 [1.1, 5.4]	1.7 [0.5, 5.4]
Location of the assault	Home environment	62	52	Reference	Reference
	Outdoor setting	20	17	1.1 [0.5, 2.5]	1.6 [0.4, 5.8]
	Other places	37	31	1.5 [0.7, 3.0]	2.8 [0.8, 9.5]
Type of assailant	Stranger (single assaults)	15	13	Reference	Reference
	Known (single assaults)	61	54	2.2 [0.9, 5.1]	5.8 [1.4, 24.9]
	Group	36	32	3.0 [1.1, 7.9]	4.2 [0.9, 19.1]
Self-defense	No/don’t know	44	35	Reference	Reference
	Yes	82	65	1.5 [0.8, 2.9]	0.9 [0.3, 2.6]
Influence of substances (victim)	No	56	44	Reference	Reference
	Yes	71	56	1.1 [0.6, 2.0]	2.5 [1.0, 6.4]
Severity of physical violence	None/mild	37	37	Reference	Reference
	Moderate/severe	64	63	5.3 [2.5, 11.5]	6.6 [2.5, 17.1]
Anal penetration	No	29	28	Reference	Reference
	Yes/attempt	76	72	1.4 [0.6, 2.8]	1.1 [0.4, 2.9]
Time lapse between assault and attending the clinic	<72 hr	98	75	Reference	Reference
	>72 hr	32	25	0.6 [0.3, 1.1]	0.7 [0.3, 1.9]

*Note.* AOR = adjusted odds ratio; OR = odds ratio; CI = confidence interval.

a Percentage with extragenital injuries, presented in column percentage.

b Estimates are adjusted for all other variables in the model.

## Discussions

The main findings of the present study showed that most male victims who attended the sexual assault clinic experienced some form of physical violence, with a relatively high number classified as moderate to severe. Assaults by multiple assailants involved more severe physical violence compared to those by a single assailant. The findings also showed a high prevalence of self-reported history of neuropsychiatric and psychiatric diagnoses, which aligns with previous clinical research (Kimerling et al., 2002; Zilkens et al., 2018). This information may be useful for mental health professionals working with this patient group. Several men also reported a history of sexual assault, though the data did not specify whether these occurred in childhood or adulthood. Some men also sought care multiple times at the clinic, indicating a risk of repeated victimization. These findings suggest that addressing repeated victimization among men and offering psychosocial support is crucial. Walker et al. (2019) emphasize the importance of prevention efforts to reduce revictimization, suggesting trauma treatment should include strategies to mitigate future risks. It is noteworthy that only a small percentage (5%) of emergency visits at Stockholm’s sexual assault center between 2015 and 2022 were made by male victims. This aligns with previous research indicating that men often avoid seeking care after sexual violence due to stigma, shame, and misconceptions about sexual violence against men (Masho & Alvanzo, 2010; Thomas & Kopel, 2023). This underrepresentation is significant given the health consequences and public health impact of sexual violence (Masho & Alvanzo, 2010).

This study found that many victims experienced anal penetration (66%), consistent with Australian and Dutch clinical data on male victims (Covers et al., 2022; Zilkens et al., 2018). Most victims also faced physical violence (71%) during the assault, with 27% encountering severe violence. Literature suggests that male victims seeking healthcare tend to experience threats, perceive danger, and sustain physical injuries during the assault (Light & Monk-Turner, 2009; Masho & Alvanzo, 2010). Furthermore, 23% of male victims were assaulted by multiple assailants, a higher prevalence compared to three international studies of male victims attending sexual assault services (Covers et al., 2022; Kane et al., 2024; Zilkens et al., 2018), but lower than in a Danish clinical sample (Larsen & Hilden, 2016). The high number of group assaults in our cohort is likely due to multiple factors, including the increased strength and violence required to subdue a man and the heightened risk of being targeted for punitive sexual assault (Kane et al., 2024; McLean et al., 2005). These factors may also contribute to the significant levels of severity found in assaults by multiple assailants. Although group assaults involved more severe physical violence, the link to extragenital injury was not significant in the adjusted regression model. This may be due to victims surrendering to avoid further harm (A. S. Möller et al., 2012) or tonic immobility, a common motor inhibition reaction during trauma (A. Möller et al., 2017). Further research on tonic immobility in male victims is recommended to inform law enforcement and healthcare.

The relationship between victims and assailants followed the same pattern as in earlier studies, with most assailants being known to the victim (Kane et al., 2024; Zilkens et al., 2018). Few victims reported being assaulted by an intimate partner, which is consistent with international clinical studies (Kane et al., 2024; Zilkens et al., 2018). This might be explained by men’s reluctance to seek care after intimate partner violence (IPV; Kane et al., 2024). It may also be due to the co-occurrence of multiple forms of violence in intimate partner assaults (Nybergh et al., 2013), leading men to seek care for other issues rather than specifically for sexual assault. Male IPV victims may also be hindered from seeking care at the clinic due to the 30-day limit for receiving care. Furthermore, an unexpectedly high number of female assailants were reported in the study. Although sexual violence by female assailants is often considered less prevalent than that by male assailants (Loxton & Groves, 2022), our findings align with literature suggesting that female assailants are more common than previously understood (Munroe & Shumway, 2020). This challenges male rape myths (Fisher & Pina, 2013; Madjlessi & Loughnan, 2024), and introduces an area of research that needs more exploration.

A surprisingly large number of victims (16%) reported amnesia during the assault, which is higher than earlier studies of male sexual assault victims (Kane et al., 2024; Zilkens et al., 2018). The uncertainty surrounding suspected sexual assault may lead to significant psychological impacts, and to our knowledge, no previous studies have specifically examined these health consequences. While alcohol overconsumption likely explains most recall deficiencies, amnesia may also be a trauma response. Substance influence was common in assaults by strangers, likely because these incidents often occur in substance-friendly settings. These findings align with an earlier study conducted at the clinic involving female victims, which identified alcohol and other substances as risk factors for sexual violence, affecting the judgment of both victim and assailant (A. S. Möller et al., 2012). Our study did not consider the assailant’s substance use, which could impact the assailant’s aggressive behavior, leading to more violent assaults and injuries.

A high proportion of the victims (65%) presented with extragenital injuries, especially on the extremities (55%), similar to an Australian clinical study (Zilkens et al., 2018). In contrast, a Danish study showed that only 38% sustained a physical injury after sexual violence (Larsen & Hilden, 2016). A possible reason for our high prevalence is that victims are motivated to seek care due to the presence of physical injury (Masho & Alvanzo, 2010). Surprisingly, the time lapse between assault and examination did not significantly affect injury detection. While literature suggests that time affects forensic examinations (A. S. Möller et al., 2012), our results indicate that male victims’ injuries are often severe enough to be evident even after 72 hrs.

### Clinical Implications

Although the victims in this research may not represent all male sexual assault victims, the study offers valuable clinical insights. It enhances understanding and awareness of male sexual victimization, the severity of violence, and assailant characteristics. Informing the public and healthcare professionals can reduce barriers for men seeking post-assault care. Additionally, the study findings can help identify specific care needs of this patient group. For instance, violence against men can be extremely severe and lead to physical injuries. These findings highlight the need to address physical injuries, conduct thorough forensic examinations, and ensure high-quality documentation of injuries, which can aid legal proceedings. Furthermore, the high severity of physical violence among men in our cohort also indicates a need for psychosocial support. This need is further supported by literature, which connects PTSD to assault characteristics and severity (Tiihonen Möller et al., 2014; Ullman et al., 2007). Moreover, a small percentage of men seek care post sexual assault, particularly in cases of IPV, emphasizing the need for health professionals to inquire about men’s experiences with violence in general practice. The study may guide prevention programs targeting specific risk profiles, such as individuals with neuropsychiatric diagnoses and psychiatric disorders. It also identifies the vulnerability of transgender and gender-diverse individuals, informing future prevention efforts. Insights into situations that increase the likelihood of encountering assailants can help develop effective prevention strategies in high-risk settings. The findings on assault characteristics among men may inform policymakers in developing regulations to protect male victims. There is a pressing need for gender-sensitive clinics in Sweden to provide equitable care to all victims, regardless of sex or gender. Currently, care provision after sexual violence is unequal across Sweden, and boys, men, and transgender individuals require increased attention and focus (The National Board of Health and Welfare, 2024).

### Strengths and Limitations

The present study has certain strengths and limitations that warrant mention. All individuals with male Social Security numbers who sought care at the clinic between 2015 and 2022 were reviewed, which reduces selection bias. However, bias remains due to the lack of data on those not seeking post sexual assault care. Forensic interviews were systematically conducted and documented, enhancing data collection. Data quality may be affected by some victim’s inability to recall the assault or reluctance to disclose details, though sensitivity analysis showed no significant differences between original and imputed data. To account for the intensity and complexity of violence, the NorAQ classification was adjusted based on the number of violent acts within each category (mild, moderate, severe). While this may more accurately reflect complex cases, it could also overestimate severity and limit comparability with studies using the original classification. Additionally, variability in the clinical assessment of mental illness, substance abuse, and prior sexual trauma may lead to reduced specificity. The centralized healthcare system in Stockholm provided diverse demographic data, but the retrospective design limited variable selection, excluding factors like migrant background, socioeconomic status, and sexual orientation. For instance, sexual orientation was not recorded during the forensic interview, which in retrospect would have been a variable of interest as men who have sex with men are at a higher risk of sexual violence (The Swedish Public Health Agency, 2019). This issue hinders the recognition of differences and similarities between groups of sexually assaulted men, which may affect the generalizability of the findings

## Conclusion

This study, with unique data from Sweden’s largest sexual assault center, aims to further understand sexual violence against men who seek post-assault care by addressing assault characteristics, violence severity, and occurrence of injury. It suggests that sexual violence against men who seek healthcare post assault can involve high levels of physical violence during the sexual assault, especially in group assaults. These findings may help identify care needs for this patient group. Additionally, the male victims in this study present a vulnerable group with high levels of mental ill-health, neuropsychiatric diagnoses, and prior exposure to sexual violence, underscoring their need for psychosocial support. Findings from this study may inform prevention programs focusing on specific risk profiles and guide the development of prevention strategies in identified high-risk settings. Despite our efforts to provide valuable evidence about sexual violence against men, research on the general population is essential for a comprehensive understanding of the phenomenon.

## Supplemental Material

sj-docx-1-jiv-10.1177_08862605251361127 – Supplemental material for Sexual Violence Against Men: A Retrospective Study on Victim Characteristics, Violence Severity, and Occurrence of Injuries Among Male Victims Attending a Sexual Assault Center Between 2015 and 2022 in Stockholm, Sweden Supplemental material, sj-docx-1-jiv-10.1177_08862605251361127 for Sexual Violence Against Men: A Retrospective Study on Victim Characteristics, Violence Severity, and Occurrence of Injuries Among Male Victims Attending a Sexual Assault Center Between 2015 and 2022 in Stockholm, Sweden by Frida M. Larsson, Anna Nielsen, Zangin Zeebari, Mariano Salazar, Anna-Mia Ekström and Anna Möller in Journal of Interpersonal Violence

sj-docx-2-jiv-10.1177_08862605251361127 – Supplemental material for Sexual Violence Against Men: A Retrospective Study on Victim Characteristics, Violence Severity, and Occurrence of Injuries Among Male Victims Attending a Sexual Assault Center Between 2015 and 2022 in Stockholm, Sweden Supplemental material, sj-docx-2-jiv-10.1177_08862605251361127 for Sexual Violence Against Men: A Retrospective Study on Victim Characteristics, Violence Severity, and Occurrence of Injuries Among Male Victims Attending a Sexual Assault Center Between 2015 and 2022 in Stockholm, Sweden by Frida M. Larsson, Anna Nielsen, Zangin Zeebari, Mariano Salazar, Anna-Mia Ekström and Anna Möller in Journal of Interpersonal Violence

sj-docx-3-jiv-10.1177_08862605251361127 – Supplemental material for Sexual Violence Against Men: A Retrospective Study on Victim Characteristics, Violence Severity, and Occurrence of Injuries Among Male Victims Attending a Sexual Assault Center Between 2015 and 2022 in Stockholm, Sweden Supplemental material, sj-docx-3-jiv-10.1177_08862605251361127 for Sexual Violence Against Men: A Retrospective Study on Victim Characteristics, Violence Severity, and Occurrence of Injuries Among Male Victims Attending a Sexual Assault Center Between 2015 and 2022 in Stockholm, Sweden by Frida M. Larsson, Anna Nielsen, Zangin Zeebari, Mariano Salazar, Anna-Mia Ekström and Anna Möller in Journal of Interpersonal Violence

sj-docx-4-jiv-10.1177_08862605251361127 – Supplemental material for Sexual Violence Against Men: A Retrospective Study on Victim Characteristics, Violence Severity, and Occurrence of Injuries Among Male Victims Attending a Sexual Assault Center Between 2015 and 2022 in Stockholm, Sweden Supplemental material, sj-docx-4-jiv-10.1177_08862605251361127 for Sexual Violence Against Men: A Retrospective Study on Victim Characteristics, Violence Severity, and Occurrence of Injuries Among Male Victims Attending a Sexual Assault Center Between 2015 and 2022 in Stockholm, Sweden by Frida M. Larsson, Anna Nielsen, Zangin Zeebari, Mariano Salazar, Anna-Mia Ekström and Anna Möller in Journal of Interpersonal Violence

## References

[bibr1-08862605251361127] Covers M. L. V. Teeuwen J. Bicanic I. A. E. (2022). Male victims at a Dutch Sexual Assault Center: A comparison to female victims in characteristics and service use. Journal of Interpersonal Violence, 37(15–16), NP14772–NP14786. 10.1177/08862605211015220 PMC9326791 33983069

[bibr2-08862605251361127] Dworkin E. R. Krahé B. Zinzow H. (2021). The global prevalence of sexual assault: A systematic review of international research since 2010. Psychology of Violence, 11(5), 497–508. 10.1037/vio0000374 34737898 PMC8562086

[bibr3-08862605251361127] Fisher N. L. Pina A. (2013). An overview of the literature on female-perpetrated adult male sexual victimization. Aggression and Violent Behavior, 18(1), 54–61. 10.1016/j.avb.2012.10.001

[bibr4-08862605251361127] Ghirardi L. Kuja-Halkola R. Pettersson E. Sariaslan A. Arseneault L. Fazel S. D’Onofrio B. M. Lichtenstein P. Larsson H. (2023). Neurodevelopmental disorders and subsequent risk of violent victimization: exploring sex differences and mechanisms. Psychological Medicine, 53(4), 1510–1517. 10.1017/S0033291721003093 37010210 PMC7618236

[bibr5-08862605251361127] Harris P. A. Taylor R. Minor B. L. Elliott V. Fernandez M. O’Neal L. McLeod L. Delacqua G. Delacqua F. Kirby J. Duda S. N. (2019). The REDCap consortium: Building an international community of software platform partners. Journal of Biomedical Informatics, 95, 103208. 10.1016/j.jbi.2019.103208 31078660 PMC7254481

[bibr6-08862605251361127] Harris P. A. Taylor R. Thielke R. Payne J. Gonzalez N. Conde J. G. (2009). Research electronic data capture (REDCap)—A metadata-driven methodology and workflow process for providing translational research informatics support. Journal of Biomedical Informatics, 42(2), 377–381. 10.1016/j.jbi.2008.08.010 18929686 PMC2700030

[bibr7-08862605251361127] Hequembourg A. L. Parks K. A. Collins R. L. Hughes T. L. (2015). Sexual assault risks among gay and bisexual men. Journal of Sex Research, 52(3), 282–295. 10.1080/00224499.2013.856836 24483778 PMC4117833

[bibr8-08862605251361127] Hiquet J. Gromb-Monnoyeur S. (2013). Men victim of sexual assault of concern into the first Emergency Medical Unit for Victims of Assaults in France. Journal of Forensic and Legal Medicine, 20(7), 836–841. 10.1016/j.jflm.2013.06.024 24112332

[bibr9-08862605251361127] Holst E. Kamra Kregert K. Viberg J. Wallin S. Westerberg S. (2023). Nationella trygghetsundersökningen 2023—Om utsatthet, otrygghet och förtroende. https://bra.se/download/18.126e8d3a18afe99a9721d6c/1696837149983/2023_Nationella_trygghetsundersokningen_2023.pdf

[bibr10-08862605251361127] Kane D. Kennedy K. M. Flood K. Eogan M. (2024). Male patient attendances at Sexual Assault Treatment Units in Ireland: An analysis of 381 cases and a comparison with female patients. Journal of Forensic and Legal Medicine, 102, 102643–102643. 10.1016/j.jflm.2024.102643 38224652

[bibr11-08862605251361127] Kimerling R. Rellini A. Kelly V. Judson P. L. Learman L. A. (2002). Gender differences in victim and crime characteristics of sexual assaults. Journal of Interpersonal Violence, 17(5), 526–532. 10.1177/0886260502017005003

[bibr12-08862605251361127] Larsen M.-L. Hilden M. (2016). Male victims of sexual assault; 10 years’ experience from a Danish Assault Center. Journal of Forensic and Legal Medicine, 43, 8–11. 10.1016/j.jflm.2016.06.007 27391940

[bibr13-08862605251361127] Light D. Monk-Turner E. (2009). Circumstances surrounding male sexual assault and rape: Findings from the National Violence Against Women Survey. Journal of Interpersonal Violence, 24(11), 1849–1858. 10.1177/0886260508325488 18981191

[bibr14-08862605251361127] Loxton A. Groves A. (2022). Adult male victims of female-perpetrated sexual violence: Australian social media responses, myths and flipped expectations. International Review of Victimology, 28(2), 191–214. 10.1177/02697580211048552

[bibr15-08862605251361127] Madjlessi J. Loughnan S. (2024). Male sexual victimization by women: incidence rates, mental health, and conformity to gender norms in a sample of British Men. Archives of Sexual Behavior, 53(1), 263–274. 10.1007/s10508-023-02717-0 37851161 PMC10794296

[bibr16-08862605251361127] Masho S. W. Alvanzo A. (2010). Help-seeking behaviors of men sexual assault survivors. American Journal of Men’s Health, 4(3), 237–242. 10.1177/1557988309336365 19706673

[bibr17-08862605251361127] McLean I. A. Balding V. White C. (2005). Further aspects of male-on-male rape and sexual assault in Greater Manchester. Medicine, Science and the Law, 45(3), 225–232. 10.1258/rsmmsl.45.3.225 16117283

[bibr18-08862605251361127] Möller A. Söndergaard H. P. Helström L. (2017). Tonic immobility during sexual assault—A common reaction predicting post-traumatic stress disorder and severe depression. Acta Obstetricia et Gynecologica Scandinavica, 96(8), 932. 10.1111/aogs.13174 28589545

[bibr19-08862605251361127] Möller A. S. Bäckström T. Söndergaard H. P. Helström L. (2012). Patterns of injury and reported violence depending on relationship to assailant in female Swedish Sexual Assault Victims. Journal of Interpersonal Violence, 27(16), 3131–3148. 10.1177/0886260512441261 22585117

[bibr20-08862605251361127] Monk-Turner E. Light D. (2010). Male sexual assault and rape: Who seeks counseling? Sexual Abuse, 22(3), 255–265. 10.1177/1079063210366271 20713746

[bibr21-08862605251361127] Munroe C. Shumway M. (2020). Female-perpetrated sexual violence: A survey of survivors of female-perpetrated childhood sexual abuse and adult sexual assault. Journal of Interpersonal Violence, 37(9–10), NP6655–NP6675. 10.1177/0886260520967137 PMC9901498 33084459

[bibr22-08862605251361127] Nybergh L. Taft C. Enander V. Krantz G. (2013). Self-reported exposure to intimate partner violence among women and men in Sweden: Results from a population-based survey. BMC Public Health, 13(1), 845. 10.1186/1471-2458-13-845 24034631 PMC3848440

[bibr23-08862605251361127] Öberg M. Heimer G. Lucas S. (2020). Lifetime experiences of violence against women and men in Sweden. Scandinavian Journal of Public Health, 49(3), 301–308. 10.1177/1403494820945072 32755350

[bibr24-08862605251361127] Peterson Z. D. Voller E. K. Polusny M. A. Murdoch M. (2011). Prevalence and consequences of adult sexual assault of men: Review of empirical findings and state of the literature. Clinical Psychology Review, 31(1), 1–24. 10.1016/j.cpr.2010.08.006 21130933

[bibr25-08862605251361127] Ports K. A. Ford D. C. Merrick M. T. (2016). Adverse childhood experiences and sexual victimization in adulthood. Child Abuse & Neglect, 51, 313–322. 10.1016/j.chiabu.2015.08.017 26386753 PMC4713310

[bibr26-08862605251361127] Sorsoli L. Kia-Keating M. Grossman F. K. (2008). “I Keep That Hush-Hush”: Male survivors of sexual abuse and the challenges of disclosure. Journal of Counseling Psychology, 55(3), 333–345. 10.1037/0022-0167.55.3.333

[bibr27-08862605251361127] Swahnberg I. M. K. Wijma B. (2003). The NorVold Abuse Questionnaire (NorAQ): Validation of new measures of emotional, physical, and sexual abuse, and abuse in the health care system among women. European Journal of Public Health, 13(4), 361–366. 10.1093/eurpub/13.4.361 14703325

[bibr28-08862605251361127] Swahnberg K. (2011). NorVold Abuse Questionnaire for Men (m-NorAQ): Validation of new measures of emotional, physical, and sexual abuse and abuse in health care in male patients. Gender Medicine, 8(2), 69–79. 10.1016/j.genm.2011.03.001 21536226

[bibr29-08862605251361127] The National Board of Health and Welfare. (2024). Att genomföra insatser för att stödja en jämlik och kunskapsbaserad hälsooch sjukvård för personer som har utsatts för sexuellt våld. https://www.socialstyrelsen.se/globalassets/sharepoint-dokument/artikelkatalog/ovrigt/2024-12-9388.pdf

[bibr30-08862605251361127] The Swedish Public Health Agency. (2019). Sexuell och reproduktiv hälsa och rättigheter (SRHR) i Sverige 2017—Resultat från befolkningsundersökningen SRHR2017. https://www.folkhalsomyndigheten.se/publikationer-och-material/publikationsarkiv/s/sexuell-och-reproduktiv-halsa-och-rattigheter-i-sverige-2017/?pub=60999

[bibr31-08862605251361127] Thomas J. C. Kopel J. (2023). Male victims of sexual assault: A review of the literature. Behavioral Sciences, 13(4), 304. 10.3390/bs13040304 37102818 PMC10135558

[bibr32-08862605251361127] Tiihonen Möller A. Bäckström T. Söndergaard H. P. Helström L . (2014). Identifying risk factors for PTSD in women seeking medical help after rape. PLoS One, 9(10), e111136. 10.1371/journal.pone.0111136 PMC4207776 25340763

[bibr33-08862605251361127] Ullman S. E. Townsend S. M. Filipas H. H. Starzynski L. L. (2007). Structural models of the relations of assault severity, social support, avoidance coping, self-blame, and PTSD among sexual assault survivors. Psychology of Women Quarterly, 31(1), 23–37. 10.1111/j.1471-6402.2007.00328.x

[bibr34-08862605251361127] Walker H. E. Freud J. S. Ellis R. A. Fraine S. M. Wilson L. C. (2019). The prevalence of sexual revictimization: A meta-analytic review. Trauma, Violence, & Abuse, 20(1), 67–80. 10.1177/1524838017692364 29333937

[bibr35-08862605251361127] Zilkens R. R. Smith D. A. Mukhtar S. A. Semmens J. B. Phillips M. A. Kelly M. C. (2018). Male sexual assault: Physical injury and vulnerability in 103 presentations. Journal of Forensic and Legal Medicine, 58, 145–151. 10.1016/j.jflm.2018.05.009 29981506

