# Supplementary material for: Sexual Violence Against Men: A Retrospective Study on Victim Characteristics, Violence Severity, and Occurrence of Injuries Among Male Victims Attending a Sexual Assault Center Between 2015 and 2022 in Stockholm, Sweden
Source: J Interpers Violence. 2025 Aug 27;41(15-16):5853–75. doi: 10.1177/08862605251361127 (PMC13373278; doi:10.1177/08862605251361127)
Supplement: sj-docx-1-jiv-10.1177_08862605251361127 – Supplemental material for Sexual Violence Against Men: A Retrospective Study on Victim Characteristics, Violence Severity, and Occurrence of Injuries Among Male Victims Attending a Sexual Assault Center Between 2015 and 2022 in Stockholm, Sweden [file sj-docx-1-jiv-10.1177_08862605251361127.docx]

**Appendix 1.** Adapted classification of minor, moderate, and severe sexual- and physical violence based on the NorVold Abuse Questionnaire (NorAQ).

| **Level** | **Classification** |
| --- | --- |
| **Sexual violence** |  |
| **Mild** | No genital contact: The assailant touched the victim's body (excluding the genitals) without sexual consent. The victim was forced to touch their own body or parts of the assailant´s body (excluding genitals).  Emotional/sexual humiliation: Exposed to verbal sexual harassment, being forced to pose sexually, being forced to watch or participate in pornography, being filmed or photographed without consent, being forced to watch sexual acts or someone naked, and more.  More than two types of mild sexual violence = moderate. |
| **Moderate** | Genital touching: The assailant touched the genitalia of the victim without consent. The victim was forced to touch the genitalia of the assailant. The victim was forced to touch his/her own genitals against their will.  More than two types of moderate sexual violence = severe. |
| **Severe** | Penetrative acts: The victim was penetrated or forced to penetrate the assailant vaginally, anally, and/or orally, either with a penis or genitalia, another body part, or an object. Attempted penetration of the vagina, anus, and mouth is also included. |
| **Physical violence** |  |
| **Mild** | The victim experienced slapping, holding, shaking, scratching, hair-pulling, spanking, biting, or acts like those mentioned.  More than one type of minor physical violence = moderate |
| **Moderate** | The victim experienced hitting with fists or objects, kicking, pushing, or other similar acts.  More than one type of moderate physical violence = severe |
| **Severe** | The victims experienced acts such as strangulation attempts, burning of the body, use or threats with a weapon or knife, or other comparable acts. |

Note: For the original version of the instrument, see the following references:

1. Swahnberg, I. M. K., & Wijma, B. (2003). The NorVold Abuse Questionnaire (NorAQ): validation of new measures of emotional, physical, and sexual abuse, and abuse in the health care system among women. *Eur J Public Health*, *13*(4), 361-366. <https://doi.org/10.1093/eurpub/13.4.361>
2. Swahnberg, K. (2011). NorVold Abuse Questionnaire for Men (m-NorAQ): Validation of New Measures of Emotional, Physical, and Sexual Abuse and Abuse in Health Care in Male Patients. *Gender medicine*, *8*(2), 69-79. <https://doi.org/10.1016/j.genm.2011.03.001>
