# Supplementary material for: Sexual Violence Against Men: A Retrospective Study on Victim Characteristics, Violence Severity, and Occurrence of Injuries Among Male Victims Attending a Sexual Assault Center Between 2015 and 2022 in Stockholm, Sweden
Source: J Interpers Violence. 2025 Aug 27;41(15-16):5853–75. doi: 10.1177/08862605251361127 (PMC13373278; doi:10.1177/08862605251361127)
Supplement: sj-docx-2-jiv-10.1177_08862605251361127 – Supplemental material for Sexual Violence Against Men: A Retrospective Study on Victim Characteristics, Violence Severity, and Occurrence of Injuries Among Male Victims Attending a Sexual Assault Center Between 2015 and 2022 in Stockholm, Sweden [file sj-docx-2-jiv-10.1177_08862605251361127.docx]

**Appendix 2:** Factors associated with the severity level of physical violence during sexual assault among male victims seeking care at a sexual assault center in Stockholm, Sweden, between 2015 and 2022.

|  |  | **Complete-case analysis** | | | **Multiple-imputation analysis***** | |
| --- | --- | --- | --- | --- | --- | --- |
|  |  |  | **Crude analysis** | **Multivariable analysis**** | **Crude analysis** | **Multivariable analysis**** |
| **Variable** | **Level** | **n (%) *** | **OR (95% CI)** | **AOR (95% CI)** | **OR (95% CI)** | **AOR (95% CI)** |
| **Age** | Adolescence (aged 13-19) | 13/80 (16%) | Reference | Reference | Reference | Reference |
|  | Young adults (aged 20-29) | 31/80 (39%) | 1.5 (0.7, 3.4) | 1.5 (0.6, 4.0) | 1.5 (0.7, 3.3) | 1.4 (0.6, 3.3) |
|  | Adults (aged 30+) | 36/80 (45%) | 2.3 (1.0, 5.3) | 2.4 (0.9, 6.6) | 2.3 (1.0, 5.2) | 2.0 (0.8, 4.7) |
| **Self-reported mental illness** | No | 31/78 (40%) | Reference | Reference | Reference | Reference |
| **(psychiatric/neuropsychiatric)** | Yes | 47/78 (60%) | 1.3 (0.7, 2.5) | 1.2 (0.6, 2.6) | 1.4 (0.8, 2.5) | 1.1 (0.5, 2.1) |
| **History of sexual** | No | 35/74 (47%) | Reference | Reference | Reference | Reference |
| **abuse** | Yes | 39/74 (53%) | 1.3 (0.7, 2.5) | 1.0 (0.5, 2.1) | 1.4 (0.8, 2.5) | 1.3 (0.6, 2.5) |
| **Location of the** | Home environment | 39/79 (49%) | Reference | Reference | Reference | Reference |
| **sexual assault** | Outdoor | 16/79 (20%) | 1.5 (0.7, 3.4) | 1. (0.3, 3.0) | 1.6 (0.7, 3.6) | 1.3 (0.5, 3.4) |
|  | Other places | 24/79 (30%) | 1.1 (0.6, 2.2) | 1.3 (0.5, 3.3) | 1.2 (0.6, 2.4) | 1.2 (0.5, 2.8) |
| **Type of assailant** | Stranger (single assailant) | 11/80 (14%) | Reference | Reference | Reference | Reference |
|  | Known (single assailant) | 37/80 (46%) | 1.2 (0.5, 2.7) | 1.5 (0.5, 4.6) | 1.2 (0.5, 2.6) | 1.2 (0.4, 3.4) |
|  | Group | 32/80 (40%) | 3.4 (1.3, 8.6) | 3.9 (1.2, 12.5) | 3.2 (1.3, 7.9) | 2.9 (1.1, 8.1) |
| **Self-defence** | No/don´t know | 15/78 (19%) | Reference | Reference | Reference | Reference |
|  | Yes | 63/78 (81%) | 1.3 (0.6, 2.6) | 1.5 (0.6, 3.6) | 1.4 (0.7, 2.6) | 1.3 (0.6, 2.6) |
| **Influence of** | No | 43/78 (55%) | Reference | Reference | Reference | Reference |
| **substances (victim)** | Yes | 35/78 (45%) | 0.7 (0.4, 1.3) | 0.7 (0.4, 1.5) | 0.7 (0.4, 1.2) | 0.7 (0.4, 1.5) |
| **Anal penetration** | No | 19/75 (25%) | References | Reference | Reference | Reference |
|  | Yes/attempt | 56/75 (75%) | 1.6 (0.8, 3.1) | 1.1 (0.5, 2.4) | 1.6 (0.7, 3.3) | - 1. (0.6, 3.0) |

* Presence of moderate/severe level of physical violence (yes), presented in the column percentage.
******All variables in the model were adjusted for.
*******All variables in the model were imputed and used as predictors. Additionally, the time-lapse between assault and attending the clinic and extra-genital injuries were used as predictors.
