# Supplementary material for: Sexual Violence Against Men: A Retrospective Study on Victim Characteristics, Violence Severity, and Occurrence of Injuries Among Male Victims Attending a Sexual Assault Center Between 2015 and 2022 in Stockholm, Sweden
Source: J Interpers Violence. 2025 Aug 27;41(15-16):5853–75. doi: 10.1177/08862605251361127 (PMC13373278; doi:10.1177/08862605251361127)
Supplement: sj-docx-3-jiv-10.1177_08862605251361127 – Supplemental material for Sexual Violence Against Men: A Retrospective Study on Victim Characteristics, Violence Severity, and Occurrence of Injuries Among Male Victims Attending a Sexual Assault Center Between 2015 and 2022 in Stockholm, Sweden [file sj-docx-3-jiv-10.1177_08862605251361127.docx]

**Appendix 3:** Factors associated with extra-genital injuries after sexual assault among male victims seeking care at a sexual assault center in Stockholm, Sweden, between 2015 and 2022.

|  |  | **Complete case analysis** | | | **Multiple imputation analysis***** | |
| --- | --- | --- | --- | --- | --- | --- |
|  |  |  | **Crude analysis** | **Multivariable analysis**** | **Crude analysis** | **Multivariable analysis**** |
| **Variable** | **Level** | **n (%) *** | **OR (95% CI)** | **AOR (95% CI)** | **OR (95% CI)** | **AOR (95% CI)** |
| **Age** | Adolescence (aged 13-19) | 21 (16%) | Reference | Reference | Reference | Reference |
|  | Young adults (aged 20-29) | 56 (43%) | 1.5 (0.7, 3.2) | 1.0 (0.3, 3.2) | 1.4 (0.7, 3.1) | 1.1 (0.4, 3.0) |
|  | Adults (aged 30+) | 53 (41%) | 2.4 (1.1, 5.4) | 1.7 (0.5, 5.4) | 2.5 (1.1, 5.8) | 1.9 (0.6, 5.4) |
| **Location of the** | Home environment | 62 (52%) | Reference | Reference | Reference | Reference |
| **assault** | Outdoor setting | 20 (17%) | 1.1 (0.5, 2.5) | 1.6 (0.4, 5.8) | 1.2 (0.5, 2.5) | 1.9 (0.6, 5.7) |
|  | Other places | 37 (31%) | 1.5 (0.7, 3.0) | 2.8 (0.8, 9.5) | 1.3 (0.7, 2.5) | 2.9 (1.0, 8.4) |
| **Type of assailant** | Stranger (single assaults) | 15 (13%) | Reference | Reference | Reference | Reference |
|  | Known (single assaults) | 61 (54%) | 2.2 (0.9, 5.1) | 5.8 (1.4, 24.9) | 2.2 (1.0, 5.0) | 5.3 (1.4, 20.4) |
|  | Group | 36 (32%) | 3.0 (1.1, 7.9) | 4.2 (0.9, 19.1) | 2.8 (1.2, 6.8) | 3.1 (0.9, 10.8) |
| **Self-defence** | No/don´t know | 44 (35%) | Reference | Reference | Reference | Reference |
|  | Yes | 82 (65%) | 1.5 (0.8, 2.9) | 0.9 (0.3, 2.6) | 1.3 (0.7, 2.4) | 1.2 (0.6, 2.5) |
| **Influence of** | No | 56 (44%) | Reference | Reference | Reference | Reference |
| **substances (victim)** | Yes | 71 (56%) | 1.1 (0.6, 2.0) | 2.5 (1.0, 6.4) | 1.1 (0.6, 1.9) | 1.6 (0.8, 3.4) |
| **Severity of physical** | None/mild | 37 (37%) | Reference | Reference | Reference | Reference |
| **violence** | Moderate/Severe | 64 (63%) | 5.3 (2.5, 11.5) | 6.6 (2.5, 17.1) | 5.4 (2.4, 12.3) | 5.6 (2.0, 15.6) |
| **Anal penetration** | No | 29 (28%) | Reference | Reference | Reference | Reference |
|  | Yes/attempt | 76 (72%) | 1.4 (0.6, 2.8) | 1.1 (0.4, 2.9) | 1.3 (0.6, 2.6) | 1.1 (0.5, 2.5) |
| **Time-lapse between** | <72 h | 98 (75%) | Reference | Reference | Reference | Reference |
| **assault and examination** | >72 h | 32 (25%) | 0.6 (0.3, 1.1) | 0.7 (0.3, 1.9) | 0.6 (0.3, 1.0) | 0.5 (0.2, 1.0) |

* Presence of extra-genital injuries, presented in the column percentage
** All variables in the model were adjusted for.
*** All variables in the model were imputed and used as predictors. Additionally, self-reported psychological and neuropsychological diagnosis and history of sexual assault were used as predictors.
