# Supplementary material for: Sexual Violence Against Men: A Retrospective Study on Victim Characteristics, Violence Severity, and Occurrence of Injuries Among Male Victims Attending a Sexual Assault Center Between 2015 and 2022 in Stockholm, Sweden
Source: J Interpers Violence. 2025 Aug 27;41(15-16):5853–75. doi: 10.1177/08862605251361127 (PMC13373278; doi:10.1177/08862605251361127)
Supplement: sj-docx-4-jiv-10.1177_08862605251361127 – Supplemental material for Sexual Violence Against Men: A Retrospective Study on Victim Characteristics, Violence Severity, and Occurrence of Injuries Among Male Victims Attending a Sexual Assault Center Between 2015 and 2022 in Stockholm, Sweden [file sj-docx-4-jiv-10.1177_08862605251361127.docx]

**Appendix 4:** Factors associated with anal injuries after sexual assault among male victims seeking care at a sexual assault center in Stockholm, Sweden, between 2015 and 2022.

|  |  | **Complete-case analysis** | | | **Multiple imputation  analysis***** | |
| --- | --- | --- | --- | --- | --- | --- |
|  |  |  | **Crude analysis** | **Multivariable analysis**** | **Crude analysis** | **Multivariable analysis**** |
| **Variable** | **Level** | **n (%)** | **OR (95% CI)** | **AOR (95% CI)** | **OR (95% CI)** | **AOR (95% CI)** |
| **Age** | Adolescence (aged 13-19) | 5 (11%) | Reference | Reference | Reference | Reference |
|  | Young adults (aged 20-29) | 19 (41%) | 2.6 (0.7, 9.1) | 2.7 (0.6, 11.1) | 2.6 (0.7, 9.6) | 3.0 (0.7, 12.1) |
|  | Adults (aged 30+) | 22 (48%) | 1.9 (0.6, 6.4) | 2.1 (0.5, 8.7) | 1.8 (0.5, 6.3) | 1.9 (0.5, 7.5) |
| **Location of the assault** | Home environment | 22 (49%) | Reference | Reference | Reference | Reference |
|  | Outdoor setting | 8 (18%) | 1.3 (0.4, 3.9) | 1.2 (0.3, 5.0) | 1.4 (0.4, 4.2) | 1.4 (0.4, 5.2) |
|  | Other places | 15 (33%) | 1.3 (0.5, 3.2) | 1.3 (0.4, 4.5) | 1.4 (0.6, 3.2) | 1.5 (0.5, 4.5) |
| **Type of assailant** | Stranger (single assaults) | 8 (18%) | Reference | Reference | Reference | Reference |
|  | Known (single assaults) | 25 (56%) | 1.0 (0.3, 3.0) | 1.1 (0.2, 4.8) | 1.0 (0.3, 3.1) | 1.1 (0.2, 5.0) |
|  | Group | 12 (27%) | 0.9 (0.3, 3.0) | 0.9 (0.2, 4.2) | 1.0 (0.3, 3.3) | 0.8 (0.2, 3.6) |
| **Self-defence** | No/don´t know | 10 (22%) | Reference | Reference | Reference | Reference |
|  | Yes | 36 (78%) | 1.2 (0.5, 3.2) | 2.1 (0.6, 6.9) | 1.5 (0.6, 4.1) | 1.5 (0.5, 4.2) |
| **Influence of** | No | 23 (52%) | Reference | Reference | Reference | Reference |
| **substances (victim)** | Yes | 21 (48%) | 0.8 (0.3, 1.8) | 0.8 (0.3, 2.2) | 0.8 (0.4, 1.7) | 0.8 (0.3, 1.9) |
| **Severity of physical** | None/mild | 20 (48%) | Reference | Reference | Reference | Reference |
| **violence** | Moderate/Severe | 22 (52%) | 1.3 (0.6, 3.1) | 1.2 (0.5, 3.1) | 1.1 (0.5, 2.4) | 1.1 (0.5, 2.5) |
| **Time-lapse between** | <72 h | 28 (62%) | Reference | Reference | Reference | Reference |
| **assault and examination** | >72 h | 17 (38%) | 1.5 (0.6, 3.5) | 1.3 (0.5, 3.6) | 1.5 (0.7, 3.3) | 1.5 (0.6, 3.6) |

Note: Only victims exposed to anal penetration or attempted anal penetration are included in the analysis.
* Presence of anal injuries (yes), presented in the column percentage
** All variables in the model were adjusted for.
*** All variables in the model were imputed and used as predictors.
